# Male awareness of prostate cancer risk remains poor in relatives of women with germline variants in DNA‐repair genes

**DOI:** 10.1002/bco2.252

**Published:** 2023-06-21

**Authors:** Vittorio Fasulo, NicolòMaria Buffi, Giuseppe Chiarelli, Giovanni Lughezzani, Monica Zuradelli, Carla Barbara Ripamonti, Monica Barile, Paolo Bianchi, Alessio Benetti, Marco Paciotti, Alessandro Uleri, Pier Paolo Avolio, Alberto Saita, Rodolfo Hurle, Federica Maura, Luca Germagnoli, Rosanna Asselta, Giulia Soldà, Paolo Casale, Massimo Lazzeri

**Affiliations:** ^1^ Department of Biomedical Sciences Humanitas University Pieve Emanuele MI Italy; ^2^ Medical Oncology and Hematology Unit IRCCS‐Humanitas Research Hospital Rozzano MI Italy; ^3^ Laboratory Analysis Unit IRCCS‐Humanitas Research Hospital Rozzano MI Italy; ^4^ Department of Urology IRCCS‐Humanitas Research Hospital Rozzano MI Italy; ^5^ IRCCS‐Humanitas Research Hospital Rozzano MI Italy

**Keywords:** *BRCA 1‐2*, DNA‐repair gene variant, genetic risk, prostate cancer, screening

## Abstract

Abstract

**Objective:**

The aim of this study is to evaluate male awareness of developing prostate cancer (PCa) in families with germline DNA‐repair genes (DRG) variants.

**Materials and methods:**

Data were collected from a prospective, monocentric cohort study. The study was conducted in a university hospital with a multidisciplinary approach to the patient (collaboration of the Departments of Oncology, Urology, Pathology, Radiology, and Medical Genetics Laboratory). We recruited healthy males, relatives of families of women with breast or ovarian cancer who tested positive for pathogenic variants (PVs) or likely pathogenic variants (LPVs) in DRGs. A dedicated PCa screening was designed and offered to men aged 35 to 69 years, based on early visits with digital rectal examination (DRE), prostate health index (PHI) measurement, multiparametric magnetic resonance imaging (mpMRI) and, if necessary, targeted/systematic prostate biopsies. The primary endpoint was to evaluate the willingness of healthy men from families with a DRG variants detected in female relatives affected with breast and/or ovarian cancer to be tested for the presence of familial PVs. The secondary endpoints were the acceptance to participate if resulted positive and compliance with the screening programme.

**Results:**

Over 1256 families, of which 139 resulted positive for PVs in DRGs, we identified 378 ‘healthy’ men aged between 35 and 69 years old. Two hundred sixty‐one (69.0%) refused to be tested for DRG variants, 66 (17.5%) declared to have been previously tested, and 51 (13.5%) males were interested to be tested. Between those previously tested and those who accepted to be tested, 62 (53.0%) were positive for a DRG variant, and all of them accepted to participate in the subsequent surveillance steps. The main limitation is that is a single‐centre study and a short follow‐up.

**Conclusions:**

All men tested positive for a DRG variants agreed to go under the surveillance scheme. However, only 31% of ‘men at risk’ (i.e., relative of a DRG variant carrier) expressed their willingness to be tested for the familial DRG variant. This observation strongly supports the urgent need to implement awareness of genetic risk for PCa within the male population.

## 1 INTRODUCTION

Prostate cancer (PCa) is the most frequent cancer in men, with 1.4 million new cases in the world in 2021.^1^ PCa accounts for approximately 15% of all cancer cases in men worldwide, and it is the most common cancer affecting men in Western countries. Globally, there are hundreds of thousands of premature deaths from PCa annually along with high morbidity, particularly related to bone metastases leading to pain, fracture, and disability.^1^, ^2^ Although there are several curative treatments such as radical surgery and radiation therapy, they can also cause adverse effects on urinary and sexual function. There is growing attention towards the need to early diagnose advanced diseases, thus avoiding underdiagnosis and undertreatment, while trying not to diagnose lower risk diseases that would likely never need lifelong care. In 2012, the US Preventive Services Task Force released a recommendation against routine use of prostate specific antigen (PSA) to screen for PCa (level of evidence [LoE]: D) because of growing concerns about overdiagnosis and overtreatment.^3^, ^4^ However, this decision was largely based on clinical trial data that have been criticized for widespread screening among control subjects and insufficient follow‐up time, and in 2017, the US Preventive Services Task Force upgraded their recommendation for men aged 55 to 69 years old (LoE: C).^5^, ^6^ Currently, the American Urological Association (AUA) and the European Association of Urology (EAU) strongly recommend screening in high‐risk populations as Afro‐Americans and men with a positive family history.^7^, ^8^

It has been estimated that 5–10% of PCa cases are related to inherited susceptibility to the disease. In particular, germline variants in *BRCA1* and *BRCA2* tumour suppressor genes are the genetic events associated with the highest risk of PCa (2.5 to 8.6 times in men <65 years), representing an independent prognostic factor for poorer outcomes.^9^, ^10^, ^11^, ^12^, ^13^ Considering the entire set of genes involved in DNA‐repair pathways, pathogenic variants (PVs) were found in approximately 8–12% of localized PCa and about 20–25% of advanced metastatic castration‐resistant PCa.^14^, ^15^ In relation to germline defects in DNA‐repair genes (DRGs), PVs were found in 11.8% of men with metastatic PCa, unselected for family history of the disease.^16^

The most common germline alterations found in patients with metastatic castrate‐resistant PCa were in *BRCA2* (5.3%), *CHEK2* (1.9%), *ATM* (1.6%), and *BRCA1* (0.9%). Other notable genes were *RAD51D* (0.4%) and *PALB2* (0.4%) genes.^17^

The association with PCa risk and treatment implications is better understood for those with variants of *BRCA2*, with emerging data supporting associations with *ATM*, *CHEK2*, *BRCA1*, *HOXB13*, *MSH2*, *MSH6* and *NBN*.^18^ PCa is set to become a much bigger burden for healthcare providers and patients in the upcoming decades, and it has been the focus of thorough research in recent years based on advanced treatment ranging from robot‐assisted surgery to focal therapy, in association with various new imaging modalities.^19^, ^20^, ^21^, ^22^

However, poor levels of PCa risk awareness have been demonstrated, and consequently, there is an unmet medical need to design acceptable and appropriate health promotion interventions and screening in healthy men with germline variants linked to high‐risk of developing PCa.^23^, ^24^, ^25^, ^26^

As highlighted in a recent study by Loeb and colleagues, there is cause for concern regarding modest social media activity and participation, as well as a lack of public awareness regarding the importance of PCa germline testing. These findings are particularly worrying given the significant advances in genetic testing for PCa that have been made in recent years.^27^

It is possible that this lack of awareness and engagement may be hindering the impact of these genetic advances, which have the potential to significantly improve early detection and treatment outcomes for PCa. It is well known the need to explore the reasons behind these trends and to identify strategies for increasing public awareness and engagement in PCa germline testing. In the meantime, it is important for healthcare professionals to continue to emphasize the relevance of genetic testing for PCa to their patients and to work towards promoting greater awareness and understanding of this critical aspect of cancer care.^27^

Genetics‐lead medicine held significant promise in improving clinical outcomes for men with familial predispositions to PCa. The use of genetics in the detection, surveillance, and management of PCa can aid in the identification of those at risk of aggressive disease, allowing for earlier intervention and personalized treatment plans.^28^, ^29^

The aim of the present study is to investigate male awareness of developing PCa in families with germline DRG variants and their willingness to follow a dedicated surveillance scheme.

## 2 MATERIALS AND METHODS

### 2.1 Study design

Data were collected from a prospective, monocentric study, starting from January 2021, designed to evaluate the sensitivity of a targeted screening in men with higher genetic risk of PCa because carriers of PVs or likely PV (class 5 or 4 according to the *ACMG/AMP* variant classification) in a DRG.^30^ The study is entirely conducted in a tertiary university hospital, throughout the collaboration of the Oncology, Urology, Pathology, Radiology, and Laboratory Departments of the Hospital with the Laboratory of Medical Genetics and RNA Biology of Humanitas University.^31^

For the purpose of the current study, only DRG PV carrier healthy men relative of women with a diagnosis of breast and/or ovarian cancer were considered for the analysis.

### 2.2 Study population

Healthy men were identified retrospectively, after reviewing the genealogical trees of all women who tested positive for a germline variant in DRGs. The term ‘healthy men’ has defined as people with no apparent history of cancer. These individuals typically have no active symptoms of disease and may have undergone routine medical screenings that have not shown any signs of PCa.

Genealogical trees were created for all patients who underwent genetic counselling at our institution for ovarian and breast cancer. They were compiled by full certified medical geneticists and included information about the patient's family members, as well as their degree of relatedness to the patient, for the purpose of medical history collection. Family trees have been used by geneticists to identify potential hereditary links to cancer within families and to assess a patient's individual risk of developing PCa.

All first/second degree male relatives of these women with DRGs PVs (followed in our institution for a history of ovarian/breast cancer) were contacted by a medical genetists, and genetic counselling or clinical visit was offered in order to perform the targeted genetic testing, according to the variant previously identified in the affected female of the family.

All individuals signed an informed consent to germline genetic screening and were instructed on the clinical implication of the result of testing.

The inclusion criteria reflect those reported in the national guidelines (Table 1).

**TABLE 1:** Inclusion criteria *BRCA* genetic analysis.

Patients affected with:	breast cancer at age < 36 years old breast cancer ‘and’ ovarian/fallopian tube or peritoneal cancer at any age bilateral breast cancer at age <50 years old triple negative breast cancer at age <60 years old male breast cancer at any age ovarian/Fallopian tube or peritoneal cancer at any age
Patients affected with breast cancer at age <50 years old and:	1 close relative with breast cancer at age <50 years old 1 close relative with bilateral breast cancer at any age 1 close relative with ovarian/Fallopian tube or peritoneal cancer at any age 1 close relative with male breast cancer at any age 1 close relative with pancreatic adenocarcinoma at any age 1 close relative with metastatic prostate cancer at high risk or intraductal/cribriform histology
Patients affected with breast cancer and:	>2 close relatives (at least 1 proband's first degree relative) with breast cancer at any age >1 close relatives (at least 1 proband' first degree relative) with ovarian/fallopian tube or peritoneal cancer at any age 1 close relative with male breast cancer at any age 2 close relatives (at least 1 proband's first degree relative) with breast, ovarian, and/or pancreatic cancer

We used a panel including all 20 DNA repair gene implicated in PCa (*ATM*, *MLH1*, *ATR*, *MRE11A*, *BAP1*, *MSH2*, *BARD1*, *MSH6*, *BRCA1*, *NBN*, *BRCA2*, *PALB2*, *BRIP1*, *PMS2*, *CHEK2*, *RAD1C*, *FAM175A*, *RAD1D*, *GEN1*, and *XRCC1*) plus three candidates to PCa predisposition genes (*HOXB13*, *EPCAM*, and *TP53*).^18^

If tested positive for familial PV, the healthy male was enrolled in the present screening for early detection of PCa. The inclusion criteria were as follows: age 35–69 years old, DRG variant predicted deleterious or suspected deleterious, ability to give informed consent according to ICH/EU GCP and national/local regulations, and compliance to follow the planned screening annual tests. The exclusion criteria were as follows: previous endoscopic surgery of the prostate, or a diagnosis of PCa, and absolute and relative contraindication to mpMRI.

The decision to set the minimum age for the screening enrolment at 35 years old was made after an internal consultation within the research group.

The institutional review board and local ethical committee (EC) approved the study (Protocol ICH‐2812, code IG 2020 ID 25027 approved by EC in 26/01/2021, official resolution in 18/02/2021). The study was granted by Associazione Italiana per la Ricerca sul Cancro (AIRC).

All patients had a telephonic or face‐to‐face medical consult where the aims, the methods, and the characteristics of the study were described. Every consult lasted 15–30 min.

### 2.3 Screening plan

**FIGURE 1:**
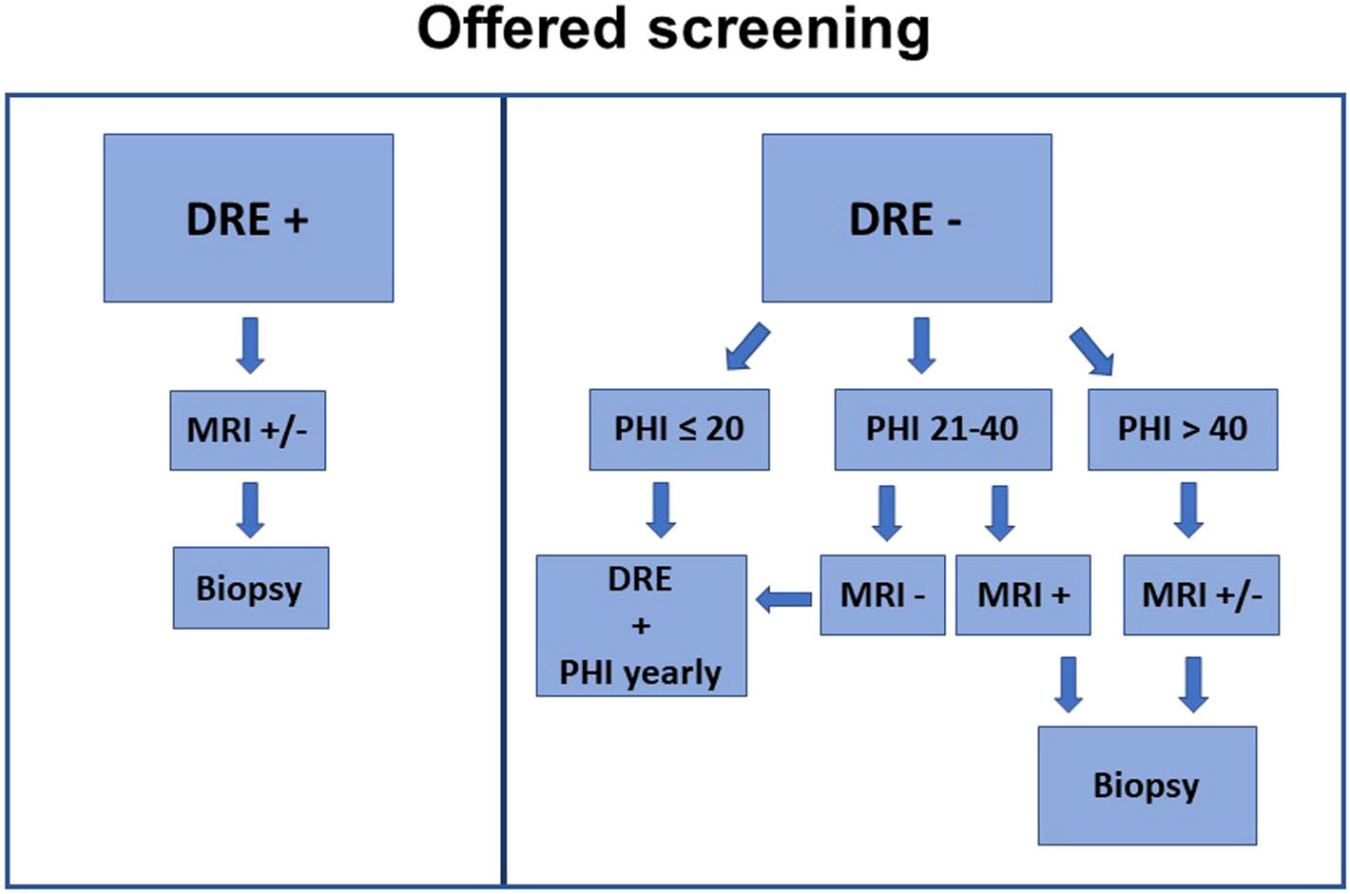
Offered screening to men resulted positive for a pathogenic variant in DNA repair genes. DRE: digital rectal examination; mpMRI: multiparametric magnetic resonance imaging; PHI: Prostate Health Index; positive mpMRI = PIRADS ≥3.

Healthy men carrying a PV were offered to be screened by calculating the Prostate Health Index (PHI), which includes Free‐PSA, Total‐PSA, −2ProPSA, and digital rectal examination (DRE) every year. In case of positive DRE, patients underwent a mpMRI, and if Prostate Imaging Reporting and Data System (PIRADS) ≥ 3 lesions, they underwent software‐assisted target fusion biopsy plus systematic biopsy or eventually systematic biopsy alone if the PIRADS was 1–2 (Figure 1).

In case of negative DRE, patients were stratified according to PHI results (Figure 1):

- If PHI ≥ 40, patients underwent an mpMRI, according to 2019 EAU guidelines, independently from the previous biopsy history, and a systematic prostate biopsy with target software assisted fusion biopsy if mpMRI showed at least a PIRADS ≥3 lesion.^7^, ^8^
- In case of PHI between 20 and 40, patients underwent a mpMRI, with target software fusion assisted biopsy plus systematic random biopsy when PIRADS ≥3; patients with a negative mpMRI (PIRADS 1–2) have been screened solely based on PHI and DRE annually.
- In the event of PHI <20, patients have been screened by DRE and PHI annually.

Considering the natural history of PCa, the primary purpose of this report is to show the clinical and pathological (PCa detection) features of the screened population.

### 2.4 Study endpoints

For the purpose of the current analysis, the primary endpoint was to evaluate the willing of healthy men from families with DRG PV/LPVs to be tested to ascertain the presence of the variant. To define the ‘willing to be tested’, a proxy for male awareness of PCa risk, the ratio between the number of men that were offered the test for *BRCA1/2* genes and men that were really tested was considered.

The secondary endpoint was to evaluate the acceptance to participate if resulted positive and compliance with screening.

## 3 RESULTS

### 3.1 Enrolment impact

We reviewed the medical records that included the genealogical trees of all breast/ovarian cancer patients who attended our Genetic Counselling Clinic from January 2016 to December 2021 and identified over 1256 families, of which 139 tested positive for PVs or LPVs in DRG. Among 139 families, we identified 378 ‘healthy’ men with age ranging between 35 and 69 years old.

**FIGURE 2:**
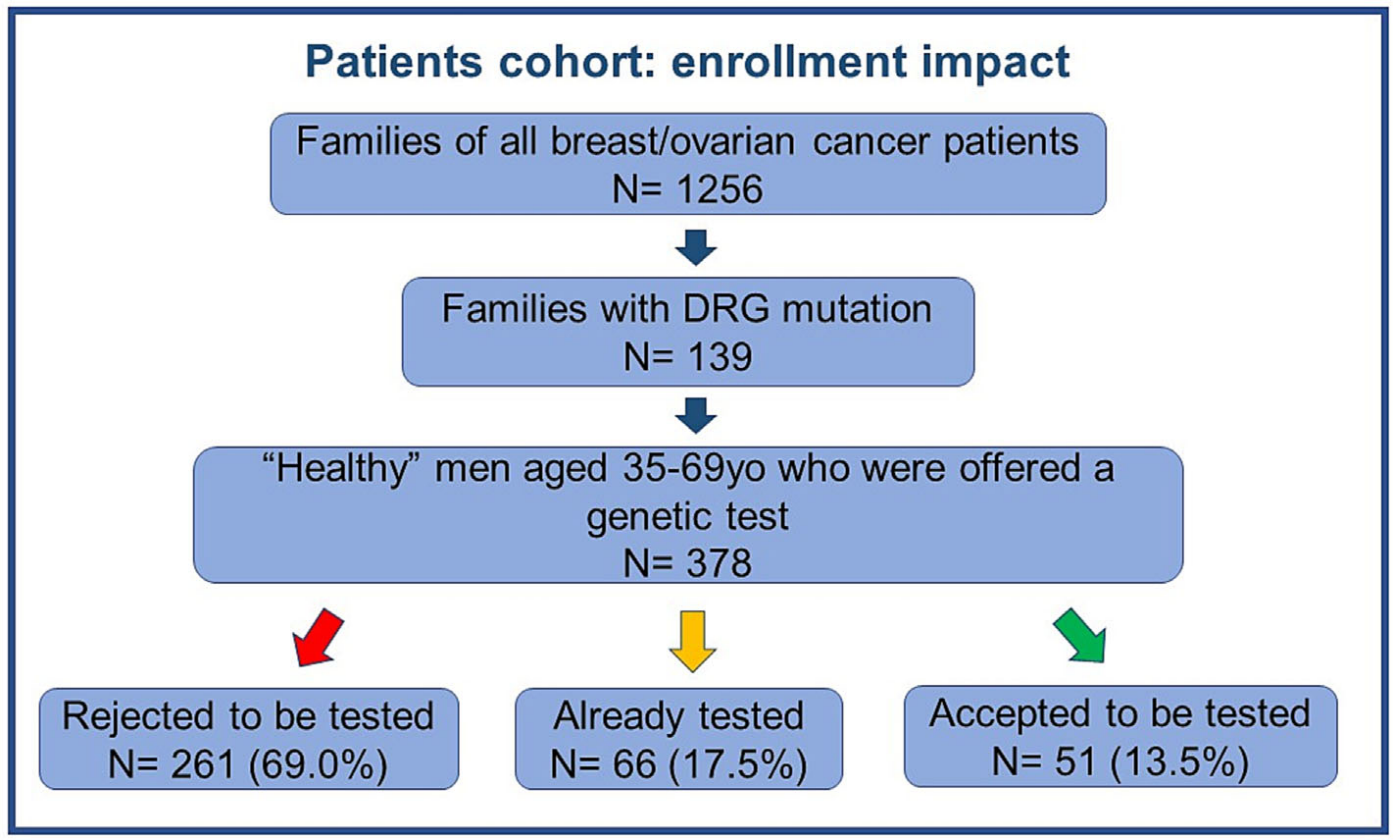
Enrolment impact of ‘healthy men’ at risk for genetic mutation. DRG: DNA repair gene.

A genetic test for detecting the familial DRG PV/LPV was offered to all healthy male subjects. Two‐hundred and sixty‐one men (69.0%) rejected to be tested, 66 (17.5%) declared to have been previously tested, and only 51 (13.5%) were interested to be tested (Figure 2).

Of those previously tested, 32 (48.5%) presented the familial PV, 19 (28.9%) were negative, and 15 (22.7%) were missed as they were unable to say or remember the result. Out of the 51 new tested men, 30 (58.8%) resulted positive.

Therefore, out of 378 selected men relatives, only 117 (31.0%) wanted to know whether they carried the familial PV, while the others were not interested or reported fear to be positive. All the patients previously tested (# 32 familial PV) and the newly tested positive (#30) who were offered to enter our dedicated PCa screening accepted to be enrolled.

Intriguingly, all of latter (100%) accepted to be enrolled in the screening and started the annual DRE and PHI.

### 3.2 Population characteristics

Of the 62 enrolled heathy men with a mean age of 52 years old (±9.45), 37 (59.7%) were carriers of PV in *BRCA2* gene, 17 (27.4%) in *BRCA1*, 3 (4.84%) in *PMS2*, 2 (3.23%) in *MSH2*, 1 (1.61%) in *MLH1*, 1 (1.61%) in *BRIP1*, and 1 (1.61%) in *ATM*.

Forty‐nine (79.0%) were born in Northern Italy, 9 (14.5%) in Southern Italy, and 4 (6.50%) in Central Italy, although the majority is living in Northern Italy: 56 (90.3%). Forty‐seven (75.8%) had at least one child, 12 (19.4%) had no child, but since just married, there were planning for a family, and only 3 (4.84%) had no child and were not married. Forty‐eight (77.4%) had a level of education equal or higher than the high school diploma of which 18 (29.0%) were graduates. Finally, only 17 (27.4%) had family history for PCa. Patients were stratified by age: 27 (43.6%) were ≤50 years old, and 35 (56.5%) were >50 years old.

### 3.3 Screening result

At the beginning of the screening program, all 62 men had negative DRE. Median PSA was 0.79 ng/mL (IQR 0.53–1.61), and median PHI was 15.0 (IQR 11.3–22.3). Forty‐four men had PHI < 20; 16 had PHI values between 20 and 40 (20 ≤ PHI < 40), and two men had PHI ≥ 40. As a consequence, 18 men underwent a mpMRI because PHI ≥ 20. The mpMRI results were distributed as follows: 17 negative (PIRADS≤2) and only one positive with a PIRADS 4 lesion. For this patient with positive mpMRI, the fusion biopsy resulted negative for PCa, and he will be screened annually. The two patients with PHI ≥ 40 had negative mpMRI; nevertheless, they underwent biopsies based on their high PHI, which was negative for both of them. Patients with 20 ≤ PHI < 40 (16 patients) underwent the mpMRI, and we obtained eight PIRADS 1, seven PIRADS 2, and one PIRADS 4 (with negative biopsies). For the 44 patients with PHI < 20, the screening will consist of an annual check‐up.

## 4 DISCUSSION

Our analysis showed that out of 378 patients' relatives of families with a known PV/LPV, only 117 (31%) decided to be tested for DRG variant. Out of these, 62 tested positive and therefore agreed to undergo screening for PCa.

Although less than 1/3 of men with siblings or parents with DRG variant accepted to know if they were carriers too, after being tested positive, their desire to be screened for an early PCa diagnosis was very high.

Furthermore, those who accepted to be tested often had an education level superior to high school diploma (77.4%), had at least one child (75.8%), and were born in Northern Italy (79.0%). Certainly, having children affects the desire to know if they are carrying variations in genes that predispose to tumours. The level of education gives a greater awareness of the medical research potential and of the scientific findings regarding early cancer diagnoses. A gap between Northern and Southern Italy screening programmes is well known, as well as a difference in advanced cancer hospitals.^32^ An imbalance in screening extension is still present, despite there was a decreasing North–South disparity trend in coverage screening programme in the last decade.^33^ Surely a well‐implemented culture on cancer screening in the territory promotes the awareness of cancer disease and the importance of early diagnosis.

In the literature, there is not much on men awareness concerning PCa diagnosis, although few studies reported that people with familiarity for PCa, even more with fatal cases, would gladly accept a genetic test, even having to pay for it.^34^ In the clinical practice of our study, the percentages were not so comforting for family members of patients with ovarian/breast cancer and DRG PVs, and only 31% underwent the genetic test. Even though they acknowledged to carry a DRG PV, thus having high‐risk to develop PCa, 100% agreed to undergo the annual urological screening.

Patients have refused genetic screening due to fear or disinterest. While we have recorded these data, it is important to note that we have not conducted a detailed analysis of the underlying reasons for this behaviour. It is possible that factors such as the patients' level of education or geographical location may be contributing to their reluctance to undergo screening. For example, individuals with lower levels of education may be less informed about the benefits of cancer screening, while those in certain regions may have cultural or religious beliefs that discourage them from seeking medical care. To better understand the factors contributing to genetic test refusal, our working group is currently exploring ways to conduct a more comprehensive analysis of this data. By identifying the specific reasons for patient reluctance to undergo screening, we can develop targeted interventions to address these issues and improve cancer screening rates in our community. We believe that likely the best way to improve our recruitment rate and spread our message across is through the help of social media.^27^, ^35^, ^36^

These data show that greater work needs to be done on social awareness of the inheritance of PCa. PCa has not yet acquired an important media coverage when compared to the ‘sister’ breast cancer, although is the first tumour for incidence in men, and it is well established how early diagnose of hereditary tumours prevents from the worst form of PCa.^37^ There is substantially less social media engagement about BRCA genes and genetic testing in PCa compared with breast cancer.^27^ Today, associations such as the AUA and the EAU strive to promote awareness campaigns aimed at stirring the social conscience among this argument, in order to anticipate the diagnosis of high‐risk tumours. As evidenced knowledge, attitudes and the benefits of PCa prevention need to spread widely in the Italian population.^23^

A number of studies have investigated the relationship between germline abnormalities, PCa screening, and risk of death from the disease. For example, a study by Pritchard et al.^18^ found that men with germline PVs in DNA repair genes had an increased risk of developing aggressive PCa, as well as a higher risk of dying from the disease. Another study by Kote‐Jarai et al.^38^ found that men with germline PVs in BRCA1 and BRCA2 genes were more likely to develop aggressive PCa and that these mutations were associated with a higher risk of death from the disease.

These findings suggest that men with germline abnormalities may benefit from PCa screening, as it could allow for earlier detection and treatment of the disease. In addition, some studies have suggested that screening may be particularly important for men with germline PVs, as they may be more likely to develop aggressive forms of PCa that are more difficult to treat. However, other studies have raised concerns about overdiagnosis and overtreatment of PCa in screened populations.^18^, ^38^, ^39^

Overall, more research is needed to determine the optimal screening strategies for men with germline abnormalities and to better understand the risks and benefits of screening in this population. However, the available evidence suggests that germline testing may play an important role in PCa screening and prevention, particularly for men with a family history of the disease.

The limitations of our study are that it is a single‐centre study, which still has low numbers of patients and a short follow‐up. COVID‐19 has certainly influenced the outcome of the study by reducing the number of prostatectomies performed in our institute.

It is important to note that the accuracy of the family tree data relied on the completeness and accuracy of the information provided by the patients and their family members. Additionally, the genealogical trees were limited to only those patients who underwent genetic counselling at our institution and may not be representative of the larger population. As a further limitation, we should acknowledge that the first counselling women attended was likely not focused on the development of PCa in their male relatives, presumably due to lack of evidence at the time. This limitation intrinsically reinforces the need to sensitize even the scientific population, which, in the context of the real world, if not completely dedicated to the topic, may not be up‐to‐date on ‘being a carrier of germinal PVs and at risk of developing PCa’. Moreover, we would like to acknowledge that the decision to start screening at 35 years old instead of 40 years old was an arbitrary decision that ended up in an internal debate.

Furthermore, screening only for male relatives of women with PVs may be a limitation; however, we wanted to test the ‘true’ awareness because when considering a relative of a PCa patient, he could be biased, because a relative of a patient is affected by the disease. In fact, we observed that they had already started a screening even without having performed the genetic test.

## 5 CONCLUSIONS

All men who tested positive for a DRG PV accepted to follow the screening. This observation strongly supports the urgent need to implement awareness of genetic risk for PCa within male population.

In addition, an accurate evaluation of the genealogical trees of breast/ovarian cancer *BRCA1‐2* patients with PVs allows the detection of men who are potentially carriers of DRG PVs and consequently enrol them in a dedicated screening, which is currently not so defined compared to the screening for breast/ovarian cancers in females. As a result, we set out to design a screening based on the combination of PHI and MRI aiming to improve the capacity of early diagnosis as well as create a potentially personalized approach.

## AUTHOR CONTRIBUTIONS

*Conception and design*: Massimo Lazzeri. *Acquisition of data*: Nicolò Buffi, Giovanni Lughezzani, Massimo Lazzeri, Monica Zuradelli, Monica Barile, Carla Barbara Ripamonti, Paolo Bianchi, Alessio Benetti, Marco Paciotti, Alessandro Uleri, Pier Paolo Avolio, Alberto Saita, Rodolfo Hurle, Federica Maura, Luca Germagnoni, Rosanna Asselta and Giulia Soldà. *Analysis and interpretation of data*: Vittorio Fasulo, Giuseppe Chiarelli and Massimo Lazzeri. *Drafting of the manuscript*: Vittorio Fasulo, Giuseppe Chiarelli and Massimo Lazzeri. *Critical revision of the manuscript for important intellectual content*: All authors. *Statistical analysis*: Vittorio Fasulo, Giuseppe Chiarelli, Massimo Lazzeri and Monica Zuradelli. *Obtaining funding*: Massimo Lazzeri and Nicolò Buffi. *Supervision*: Paolo Casale and Rosanna Asselta.

## CONFLICT OF INTEREST STATEMENT

All authors declare no conflict of interest.
